# Proof of an optimized salicylic acid paste-based treatment concept of ulcerative M2-stage digital dermatitis lesions in 21 dairy cows

**DOI:** 10.1371/journal.pone.0269521

**Published:** 2022-06-09

**Authors:** Maher Alsaaod, Tim K. Jensen, Lea Miglinci, Corinne Gurtner, Sabine Brandt, Jeanette Plüss, Eveline Studer, Adrian Steiner

**Affiliations:** 1 Clinic for Ruminants, Vetsuisse Faculty, University of Bern, Bern, Switzerland; 2 Center for Diagnostic, Technical University of Denmark, Kongens Lyngby, Denmark; 3 Research Group Oncology, Equine Clinic of Surgery, Department of Companion Animals and Horses, University of Veterinary Medicine, Vienna, Austria; 4 Department of Infectious Diseases and Pathobiology, Institute of Animal Pathology, Vetsuisse Faculty, University of Bern, Bern, Switzerland; Michigan State University, UNITED STATES

## Abstract

The efficacy of salicylic acid paste (SA) in the treatment of ulcerative bovine digital dermatitis (BDD) was assessed by combining clinical and histopathological analyses with molecular biological techniques. The latter were conducted in a blinded manner to reach maximum objectivity. Prior to treatment, M2-stage BDD lesions (n = 26, diagnosed in 21 dairy cows) exhibited ulceration, with severe perivascular, chronic, lymphoplasmacytic dermatitis and extensive keratinolysis being noted in most cases. Pretreatment biopsy samples (n = 12) followed by povidone-iodine ointment under bandage for one week before administration of SA paste were tested positive for *Treponema* spp. by blinded PCR and fluorescent in situ hybridization (FISH). Subsequent treatment consisted of application of SA and bandaging at weekly intervals until lesions had completely resolved. The treatment duration ranged between 2 and 4 weeks. Complete healing was achieved in 100% of cases, with 2/21 animals requiring a second round of treatment upon disease reoccurrence. Importantly, only 3/26 biopsies taken from previously affected sites still tested positive by *Treponema* PCR, and in another biopsy, the outermost layers of the stratum corneum scored weakly positive by *Treponema*-specific FISH. None of these *Treponema* DNA-positive biopsies showed signs of ulceration. One case exhibited focal keratinolysis. Positive PCR or FISH in these cases may have arisen from DNA traces of dead bacteria or environmental contamination during biopsy harvesting. To our knowledge, this is the first study on blinded molecular biological monitoring of the therapeutic efficacy of SA with respect to treponemal infection, and on complete BDD M2-stage remission in all animals achieved by SA treatment according to an optimized protocol. Although the etiology of BDD is considered as multifactorial, our data further support the concept that treponemes have a decisive role in BDD pathogenesis.

## Introduction

Bovine Digital Dermatitis (BDD) is an infectious disease in cattle that clinically corresponds to an inflammatory dermatitis of the digital skin, mainly at the plantar aspect of the feet between the heels [1]. Acute BDD is associated with considerable pain and lameness, thus constituting a serious health and welfare issue. In addition, disease has a negative impact on the dairy industry, as it results in high treatment costs, decreased milk yield and reduced fertility in affected animals [2–4]. BDD is prevalent in many countries with dairy farming, and often endemic, with e.g. a farm prevalence of up to 94% reported for North America [5]. BDD is currently regarded as multifactorial disease driven by environmental and management factors as well as bacterial infection. Anaerobic spirochetes, notably *Treponema* spp. are consistently associated with the onset and progression of BDD lesions [6–9]. In acute lesions, *Treponema* spp. are present as motile spirals, while they are encysted in chronic lesions [6]. Histopathologically, BDD lesions are characterized by epithelial changes including hyperplasia, degeneration and necrosis as well as by visible spirochetal colonization of the stratum corneum. In severe cases, spirochetal infection can involve the whole epidermis and induce dermal inflammation [10, 11].

In recent years, BDD showed an increasing tendency to become a chronic disease [12, 13]. Recurrence is frequently seen, indicating that natural immunity against spirochetes is not achieved, and that state-of-the-art prevention and control measures are not sufficiently effective [14]. Once established in a population, complete elimination of BDD seems rather impossible [15]. Topical treatment of individual BDD-affected cows, especially with antimicrobials such as chlortetracycline spray, is the most commonly used therapeutic strategy [16, 17]. However, there is growing interest in replacing antibiotics by alternative substances in view of the risk of residual antibiotic traces in milk and meat, and the increasing problem of antibiotic resistances [18–20].

In the past few years, salicylic acid (SA) powder or paste have emerged as nonantibiotic alternative in the treatment of BDD and interdigital phlegmon. SA is a 2-hydroxybenzoic acid with bactericidal and anti-inflammatory properties. Commercially available SA paste is formulated with methylsalicylate, a stimulator of blood circulation and phagocytosis [21]. SA primary acts through keratolysis, leading to dissolution of the intercellular cement within the stratum corneum [22]. In addition, SA abrogates bacterial growth by affecting cell wall and cell surface components [23], and by down-regulating fibrinogen, fibronectin, and α-hemolysin factors involved in bacterial replication [24, 25].

There are several studies providing evidence that SA is effective in BDD therapy. However, reported BDD remission rates greatly vary depending on SA administration protocols and treated BDD stages. In most studies, therapeutic monitoring was based on repeated clinical evaluation of lesions [18, 26–29].

BDD is commonly diagnosed by clinical examination [30, 31], providing no information on spirochetal infection and inflammation levels. In-depth analyses are required to address these key indicators of therapeutic success, as exemplarily carried out by Capion et al. [18]. The latter addressed the performance of SA also by repeated histopathological analysis of lesional biopsies to study the correlation between clinical BDD stages and spirochetal infection levels. Of note, different phylotypes of *Treponema* spp. can also reside deep in the stratum spinosum and the dermal papillae of BDD lesions [11]. In addition, *Treponema* DNA was also identified in clinically BDD-free skin and healing stages of BDD following topical treatment with oxytetracycline using PCR [12, 32]. These findings indicate that BDD treatment efficacy studies should ideally include molecular biological analyses allowing for highly sensitive detection of intralesional spirochetes.

In the current study, we hence combined clinical and histopathological analyses with *Treponema*-specific PCR and fluorescent in situ hybridization (FISH) to evaluate the effect of repeated local administration of SA under bandage in the treatment of ulcerative M2-stage BDD lesions. The combination of molecular biological analyses allows for precise monitoring of the response of M2-stage BDD lesions to repeated treatment with SA paste under bandage, with respect to elimination of intralesional colonization by treponemes after complete clinical healing.

Our expected outcomes were that (i) the optimized salicylic acid paste-based treatment concept would allow for complete clinical healing of M2-stage lesions in all study cases, and that (ii) signs of colonization of the epidermal layers underneath the stratum corneum by Treponema spp. would be absent after clinical disease remission.

In the herein presented study, we evaluated the efficacy of an optimized SA-based treatment protocol with respect to (i) complete clinical disease remission, and, importantly, (ii) the elimination of signs of intralesional colonization by treponemes. This was achieved by an approach combining clinical examinations at regular intervals with histopathological and molecular biological analyses of biopsies taken before treatment and following complete clinical disease remission.

## Materials and methods

### Ethical statement

The study protocol was approved by the animal experimentation committee of the cantons of Bern and Fribourg, Switzerland (permission # BE 20/19+).

### Animals and housing

The study involved 21 dairy cows kept at an agricultural school (Farm A, Inforama Rütti, Zollikofen, Switzerland; n = 12) and a Holstein-breeding farm (Farm B, Plaffeien, Switzerland; n = 9) between May 2019 and April 2021. The two farms were selected by convenience from all that participated in the routine herd health service offered by the Clinic for Ruminants, Vetsuisse Faculty, University of Bern, Switzerland. At both farms, the cows were kept in a loose housing system with straw-bedded cubicles and daily access to pasture during the grazing season (April to October; farm A). At farm B, the cows moved to the alpine meadow in summer. The walking and feeding alleys were made of plain rubber and slatted concrete in farm A, and plain concrete in farm B. The cows were milked twice a day and had free access to a total mixed ration and a water trough. At the entrance into the study, the mean age of the cows was 4.6 years (range: 2.33–8.77 years), the mean days in milk was 134 (12–587). The breeds involved were Holstein Friesian (n = 11), Red Holstein (n = 5), Swiss Fleckvieh (n = 4), and cross-breed (n = 1). Functional claw trimming was performed twice a year according to a standardized protocol using an angle grinder [33, 34]. Both farms did not use any disinfecting footbath.

### Clinical foot examination

All fore and rear feet inspections were performed at weekly intervals in the milking parlor during milking (farm A), or with the cows in headlocks (farm B), using the BDD scoring method adapted from Relun et al. [31]. In brief, all feet were cleaned with water from the hose. Then, a mirror equipped with an LED light was used to thoroughly inspect each foot.

BDD lesions were scored by a trained and experienced practitioner (study author MA) according to the 5-point M-stage scoring system established by Döpfer et al. [7] and extended by Berry et al. [35]. Lesions were scored as follows (“M” referring to Cheli & Mortellaro’s initial description of the disease), with M1 describing a small (<2 cm cross section) focal active lesion, M2 a larger lesion (>2 cm cross section) with an active ulcer, M3 a healing, scabbed lesion, M4 a chronic stage of infection characterized by hyperkeratosis or surface proliferation, M4.1 a chronic stage with small active M1 lesions, and M5 a healed stage showing no clinical signs of pre-existing lesions (= normal skin). Furthermore, the M2-stage lesions were classified according to the International Committee for Animal Recording (ICAR) Claw Health Atlas (Digital Dermatitis Stages, Appendix 1) into ulcerative or proliferative M2-stage lesions [36].

Cows with signs of M2-stage lesions were further inspected in the trimming chute, the results of which served as reference for the identification of BDD. All examined feet were scored, the maximal extension of the lesions measured on site and photographed. Cows with ulcerative M2-stage lesions located dorsally/and or plantarly/palmarly in the digital skin between the digits with or without involvement of the interdigital skin were included in the study. At the beginning of the study, the BDD prevalence was estimated at 63.6% (farm A) and 45.8% (farm B), respectively.

### Experimental procedures, sample collection and treatment

A total of 26 ulcerative M2-stage lesions were subject of this study (**Table 1 and S1 Dataset**). In a preceding pilot study, application of SA directly onto the biopsy site had led to phlegmon formation in two animals. For the main study, it was therefore decided to collect pretreatment biopsy samples from a randomly selected subset of cattle only (n = 12) and to apply povidone iodine ointment (Betadine® disinfectant ointment ad us. Vet., Covetrus AG, Lyssach, Switzerland) for one week under bandage before administering SA paste. When no pretreatment biopsy was taken (n = 14), treatment was initiated with SA under bandage. After scoring and before sampling, the cows in the trimming chute were subjected to interdigital anesthesia. The plantar area of the feet (heels up to the dew claws) was clipped, meticulously cleaned and thereafter disinfected, alternatingly using 1%-povidone-iodine solution and 70% alcohol wipes (3 times of each). Interdigital locoregional anesthesia at the level of the distal P1 was administered using 20 ml of 2% Lidocaine (Streuli Tiergesundheit AG, Uznach, Switzerland). After 10 min, the temporary loss of sensitivity of the lesion to tactile stimulus was confirmed using clamping forceps. Then, one biopsy (B1) from the center of the lesion was collected in 12 cases using a sterile biopsy punch (4 mm in diameter with a maximum depth of 7 mm), and transferred directly to a sterile petri dish. After B1 collection, povidone-iodine ointment was applied onto the biopsy site, followed by application of a bandage that remained in place for one week, to prevent bacterial infection at the biopsy site. The SA paste (Novaderma ad us. vet., Paste, Streuli Tiergesundheit AG, Uznach, Switzerland) was subsequently applied (~ 4 mm thickness; 1 g containing 660 mg SA and 7.7 mg methylsalicylate) to the pre-cleaned lesion (initial cleaning of the area with a povidone iodine soap) including a border of clinically healthy skin of 5 mm. In addition, Milker’s fat cream (Eutra Tetina vet., Interlac, Puidoux, Switzerland) was applied to unaffected surrounding skin and the interdigital space to protect these areas from the keratolytic effect of the SA paste. Then, lesions were kept under bandage for seven days [37]. Subsequently, bandages were removed, the lesions were washed with water, loose skin remnants were carefully removed, and then the affected areas were dried with a towel before scoring and taking photographs. In all cases (n = 26), SA paste was applied under bandage at weekly intervals until lesions had completely resolved (M5-stage).

**Table 1:** Treatment duration (weeks) until complete clinical healing and results of bacteriological, histopathological evaluation and fluorescent in situ hybridization of biopsies obtained before treatment (M2-stage lesions) and after complete clinical healing (M5-stage lesions).

^1^Animal	^2^Limb	^3^Treatment duration (weeks)	Bacteriological results				Histopathological results						FISH results			
			^4^TT PCR		^5^Amplified sequence: [GenBank accession no.] *best match* (identity in %)		^6^Histopathology/keratinolysis score (0–3)		^7^Ulceration (0–1)		^8^Spirochete load (0–3)		^9^*Treponema* spp. Score (0–3)		*Treponema* phylotypes	
			M2	M5	M2	M5	M2	M5	M2	M5	M2	M5	M2	M5	M2	M5
1	RL	2	+	-	[KR025809] *T*. *medium* (85)	-	3/3	1/0	1	0	2	0	3	0	*T*. *phagedenis*, *T*. *pedis*, *T*. *medium*, *T*. *refringens*	^£^x
	RR	2	na	-	na	-	na	1/0	na	0	na	0	na	0	na	x
2	RL	2	+	-	[CP054692 *] T*. *phagedenis* (98)	-	2/2	1/0	1	0	2	0	3	0	*T*. *phagedenis*, *T*. *pedis*, *T*. *medium*, *T*. *refringens*	x
3	RR	2	+	-	[CP054692] *T*. *phagedenis* (84)	-	2/2	1/0	1	0	2	0	3	0	*T*. *phagedenis*, *T*. *pedis*, *T*. *medium*, *T*. *refringens*	x
4	RL	2	+	-	[AM942448] PT4 (94)	-	2/2	1/0	1	0	0	0	3	0	*T*. *phagedenis*, *T*. *pedis*, *T*. *medium*, *T*. *refringens*	x
5	RR	2	+	-	[AM942448] PT4 (91)	-	3/3	0/0	1	0	1	0	3	0	*T*. *phagedenis*, *T*. *pedis*, *T*. *medium*, *T*. *refringens*	x
	RL	2	+	-	[CP054692] *T*. *phagedenis* (91)	-	2/2	0/0	1	0	0	0	3	0	*T*. *phagedenis*, *T*. *pedis*, *T*. *medium*, *T*. *refringens*	x
6	RR	2	+	-	[CP054692] *T*. *phagedenis* (91)	-	2/2	1/0	1	0	1	0	3	0	*T*. *pedis*, *T*. *medium*, *T*. *refringens*	x
7	RL	3	^10^na	-	na	-	na	0/0	na	0	na	0	na	0	na	x
8	RR	2	na	-	na	-	na	1/0	na	0	na	0	na	0	na	x
9	RR	2	na	-	na	-	na	0/0	na	0	na	0	na	0	na	x
10	RR	2	na	-	na	-	na	0/0	na	0	na	0	na	0	na	x
11	RL	2	na	+	na	[AM942447] PT3 (98)	na	1/0	na	0	na	0	na	0	na	x
12	RR	3	na	-	na	-	na	2/0	na	0	na	0	na	0	na	x
13	*RR	3	+	-	[JN713413] Canine oral T. (93)	-	2/3	1/0	1	0	0	0	3	0	*T*. *phagedenis*, *T*. *pedis*, *T*. *medium*, *T*. *refringens*	x
14	RL	3	+	+	[JN713413] Canine oral T. (97)	[KR025809] *T*. *medium* (98)	2/3	2/0	1	0	1	0	3	0	*T*. *phagedenis*, *T*. *pedis*, *T*. *medium*, *T*. *refringens*	x
15	RL	3	+	-	[JN713413] Canine oral T. (96)	-	2/3	1/0	1	0	0	0	3	0	*T*. *phagedenis*, *T*. *pedis*, *T*. *medium*	x
16	RL	3	na	-	na	-	na	1/1	na	0	na	0	na	0	na	x
	*RR	3	+	-	[AM980447] PT8 (99)	-	2/3	1/0	1	0	0	0	3	0	*T*. *phagedenis*, *T*. *pedis*, *T*. *medium*	x
17	RR	3	+	+	[JN713413] Canine oral T. (96)	[AF426101] *T*. *refringens* (87)	2/2	1/1	1	0	0	0	3	0	*T*. *phagedenis*, *T*. *pedis*, *T*. *medium*, *T*. *refringens*	x
18	RR	2	na	-	na	-	na	1/0	na	0	na	0	na	0	na	x
19	FL	2	na	-	na	-	na	1/0	na	0	na	0	na	0	na	x
	RL	3	na	-	na	-	na	1/0	na	0	na	0	na	0	na	x
	^§^RR	4	na	-	na	-	na	1/0	na	0	na	0	na	0	na	x
20	^§^RL	3	na	-	na	-	na	0/0	na	0	na	0	na	1	na	NI
21	RR	2	na	-	na	-	na	2/0	na	0	na	0	na	0	na	x

^1^ Animal identification number: from 1 to 12 (farm A) and 13 to 21 (farm B)

^2^ Foot: FL = fore left, RL = rear left, RR = rear right

^3^ Treatment duration from application of the first bandage (M2-stage lesions) until complete clinical healing (M5-stage lesions). The DD lesions were scored according to the 5-point scale by Dopfer et al. [7] and extended by Berry et al. [35]. (M2-stage): active ulcerative M2-stage lesions were defined as >2 cm cross section and M5-stage lesions as healthy skin with no clinically visible lesions (normal skin).

^4^ TT PCR: detects *Treponema* spp. as well *Tannerella* spp. by using universal *Treponema* primers 5′/3′ TT (+: presence and–: absence)

^5^ GenBank accession number for the 16S rRNA gene; best match of the isolates (identity %)

^6^ Degree of keratinolysis was scored as: 0 = no changes; 1 = focal; 2 = moderate; 3 = extensive. Degree of chronic lympho-plasmacytic perivascular dermatitis was scored as: 0 = absent; 1 = mild; 2 = moderate; 3 = severe. The histological findings were scored according to Read and Walker [40] and modified by Klitgaard et al. [11].

^7^ Ulceration was scored as 0 = absence and 1 = presence.

^8^ The number of spirochetes in the Warthin-Starry stain was scored as 0 = none visible; 1 = minimal amount; 2 = moderate amount; 3 = high amount.

^9^ Fluorescent in situ hybridization for the detection of *Treponema* spp.: 0 = no hybridization, 1 = sparse hybridization, 2 = moderate hybridization, and 3 = strong hybridization.

^10^ na = not applicable; no biopsy was collected and salicylic acid was applied directly onto the initial M2-stage lesions; NI: positive, but phylotypes not identified;* Re-treatment because of recurrence 15 and 22 weeks after complete clinical healing, respectively; ^§^ The dorsal aspect of the foot was also affected.

After complete clinical healing (M5-stage) was reached, final biopsies were taken as previously described for B1. Two biopsies (B2) from 2 different sites of the former M2-stage lesion were taken using sterile biopsy punches (3 mm in diameter). Subsequently, a povidone-iodine ointment bandage was applied for one week as previously described. If the dorsal aspect of the feet was also affected (animals #16, #19 and #20; Table 1), the second B2 biopsy was taken from the former dorsal M2-stage lesion. One animal (# 17) also exhibited M2-associated interdigital hyperplasia (IH; IH+M2). In this case, no biopsy was taken from the interdigital skin, and the treatment of the IH+M2 lesion was clinically evaluated only [38]. One week after B2 collection, bandages were removed and feet evaluated in the trimming chute for any signs of inflammation. This clinical evaluation was repeated at weeks 4, 15 and 22 after B2 collection. Treated cows were kept with their herdmates in the main stall.

Bandaging was carried out as follows: an ergonomic, padded and water-repellent compress (ITIN+HOCH GmbH, Liestal, Switzerland) was applied to the affected area. A cotton wool roll was wrapped around the distal extremity in two circular layers followed by application of a cohesive tape (Cohesive bandages, Covetrus AG, Lyssach, Switzerland) in a figure of eight, integrating the interdigital space. Finally, an adhesive tape (Tesa, Covetrus AG, Lyssach, Switzerland) was applied to increase the moisture repellent characteristics of the bandage, taking care not to apply it directly onto the skin.

Wilcoxon rank sum test was used to determine the difference of treatment duration between cows with pretreatment biopsy collection and administration of PVP-iodine ointment and cows without pretreatment biopsy collection and without administration of PVP-iodine ointment, using the statistics package NCSS (NCSS LLC, Kaysville, UT; www.ncss.com/).

### Biopsy sample processing

Each individual biopsy collected before (B1 samples) and after treatment (B2 samples) was longitudinally dissected on a sterile petri dish using sterile forceps and #11 sterile scalpel blades.

For PCR screening, the outermost portion of the epidermis (~ 1 mm) of one half of the biopsy specimen was removed and discarded, and the remaining portion was immediately transferred to an Eppendorf tube and stored at –20°C until further processing.

For histological examination, the second half of each biopsy was fixed in 10% neutral buffered formalin for 24 h, then trimmed, embedded in paraffin, and sectioned at 4 μm. All sections were routinely stained with hematoxylin and eosin (HE) and with a Warthin-Starry stain (WS-stain) [39] for visualization of spirochetes. B2 samples were pooled for PCR screening and separately evaluated for histological analyses by considering the maximum score.

### Histopathological scoring

The histopathological scoring of sections was performed as described by Read and Walker [40] and modified by Klitgaard et al. [11] to allow for classification of epidermal and dermal changes. BDD-associated changes commonly include i) focally circumscribed hyperplastic epidermis with or without parakeratotic papillomatous proliferation, ii) loss of the stratum granulosum, and/or iii) dermal inflammation. Scoring also took respective degrees of keratinolysis and chronic dermatitis into account. Keratinolysis was graded as follows: 0 = none; 1 = focal, 2 = multifocal, 3 = extensive. Chronic dermatitis was classified as follows: 0 = no changes present; 1 = mild perivascular, chronic, lymphoplasmacytic dermatitis; 2 = moderate perivascular, chronic, lymphoplasmacytic dermatitis; 3 = severe perivascular, chronic, lymphoplasmacytic dermatitis. The number of spirochetes visualized by WS-silver-staining was semi-quantitatively categorized as: 0 = none visible; 1 = minimal number, 2 = moderate number, 3 = high number of spirochetes.

#### PCR screening for *Treponema* DNA

B1- and B2-samples were transferred to the laboratory of the Research Group Oncology (RGO), University of Veterinary Medicine, Vienna, Austria, where PCR analyses were carried out in a blinded manner. Whole DNA was extracted from numbered biopsy aliquots using a commercial DNeasy Blood and Tissue kit (Qiagen, Hilden, Germany) according to instructions of manufacturer. PCR compatible quality of DNA isolates was confirmed by standard β-actin PCR as previously described [41]. Subsequently, DNA extracts were screened for the presence of treponemal DNA using consensus *Treponema* primers 5′/3′ TT [42] according to an optimized protocol [43]: PCRs were carried out in 0.65 ml MμlTI Ultra PCR tubes (Sorenson™ BioScience, Inc., Salt Lake City, Utah, USA), each containing 9.5 % DMSO (Sigma-Aldrich, Vienna, Austria), 5 μl 10x PCR buffer (Roche, Vienna, Austria; 10 mM Tris/HCl pH 8.3, 50 mM KCl, 1.5 mM MgCl_2_), 1.5 mM of each dNTP (Roche) 100 pmol sense and antisense primer (Eurofins, Vienna, Austria), 1 μl DNA template and 1 U Taq polymerase (Roche) in a volume of 50 μl. The amplification program consisted of seven touch-down cycles [92°C for 30 s/65–56°C for 45 s (−1.5°C per cycle)/72°C for 45 s], followed by 40 standard cycles (92°C for 30 s/56°C for 45 s/72°C for 45 s) and a final elongation step at 72°C for 5 min.

For each PCR, the following controls were included: a *Treponema* DNA-positive BDD DNA isolate as the positive control, confirmed *Treponema*-free equine skin DNA as the negative control, and sterile water as the no-template control (ntc).

Amplification products (16 μl) were analyzed by electrophoresis on 1.5% Tris-acetate-EDTA agarose gels and visualized by ethidium bromide staining, with a GeneRuler 100 bp DNA ladder (ThermoScientific, Vienna, Austria) serving as molecular weight marker. Amplicon aliquots (34 μl) of anticipated size were gel-purified using a QIAex II gel extraction kit (Qiagen) according to the manufacturer’s instructions and then subjected to direct bidirectional sequencing (Eurofins Genomics) using *Treponema* primers 5′/3′ TT with a dilution of 1:10.

After aligning the 3’ end and the 5’ end sequences, only the matching part was identified via BLAST alignment (https://blast.ncbi.nlm.nih.gov/Blast.cgi). This resulted in identification of the most abundant *Treponema* DNA sequence per sample.

### FISH scoring

For blinded FISH analysis, formalin-fixed, paraffin-embedded (FFPE) biopsies were transferred to the Technical University of Denmark (TKJ). Serial 4-μm sections were prepared, mounted on Epredia™ SuperFrost Plus™ adhesion slides (Fisher Scientific, Roskilde, Denmark) and hybridized as previously described by Rasmussen et al. [44]. The oligonucleotide probes used in this study are listed in S1 Table and included probes specific for the domain Bacteria, the genus *Treponema*, and four different *Treponema* phylotypes, i.e. *T*. *pedis*, *T*. *phagedenis*, *T*. *medium*, and *T*. *refringens*. The probe for the domain *Bacterium* was 5’-labeled with fluorescein isothiocyanate (FITC), all other probes were 5’-labeled with the isothiocyanate derivative Cy3 (Eurofins Genomics, Ebersberg, Germany). The probe for the domain *Bacterium* was used on all slides in combination with one other probe for individual bacterial species. In brief, the slides were mounted in a Sequenza slide rack (Thermo Shandon, Cheshire, United Kingdom) and the hybridization was carried out at 45˚ C for 16 h. After hybridization, the slides were washed in washing buffer, rinsed in water, air dried, and mounted in Vectashield (Vector Laboratories Inc., Burlingame, CA, USA) for fluorescence microscopy.

For each of the applied *Treponema* probes, the hybridization signal was scored from 0 to 3 according to Klitgaard et al. [11]: 0 = no hybridization, 1 = sparse hybridization, 2 = moderate hybridization, and 3 = strong hybridization.

## Results

### Clinical findings

**Fig 1:**
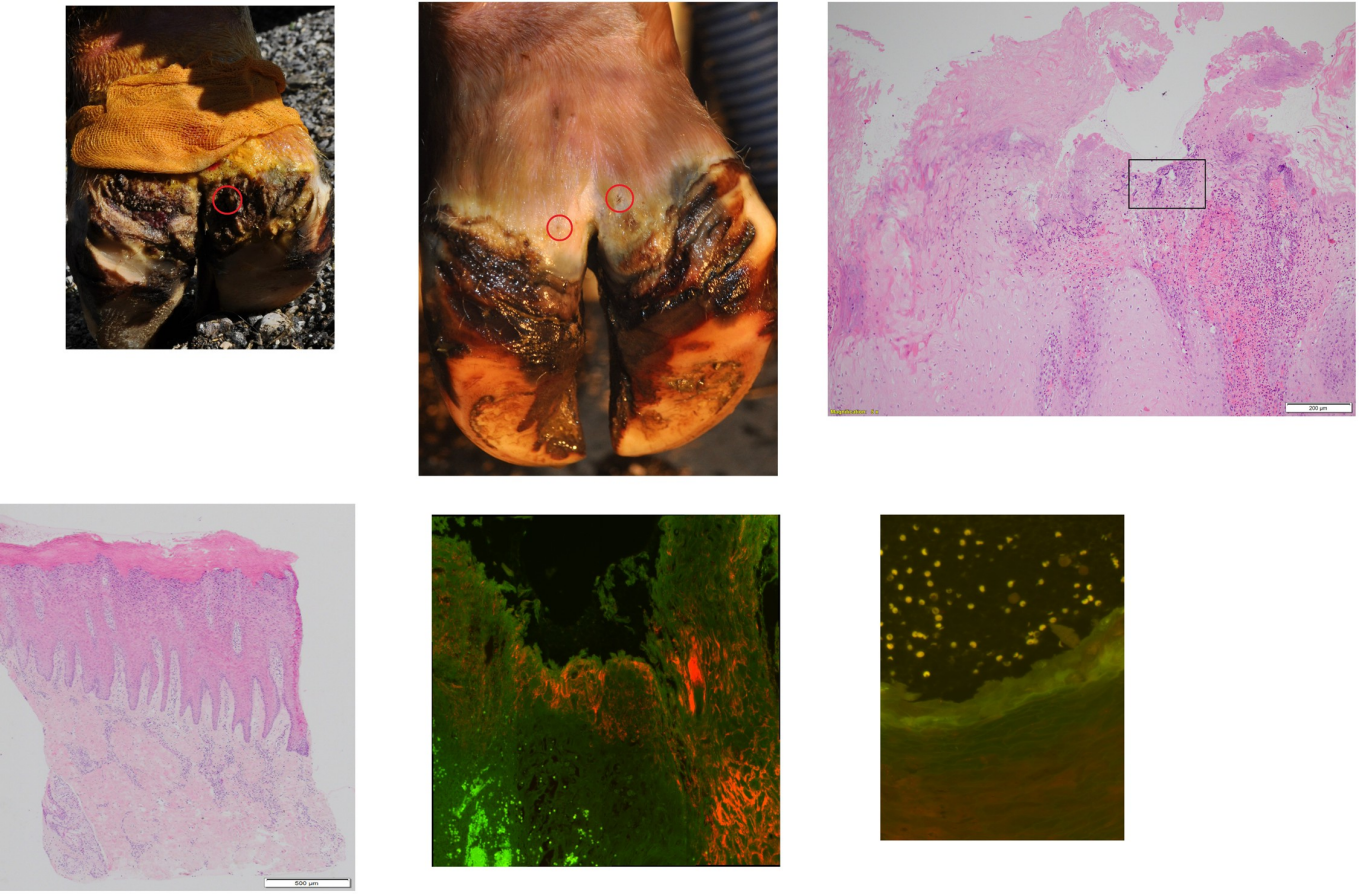
Clinical, histological appearance and fluorescent in situ hybridization of biopsies of a dairy cow (cow #13; age: 27 months) with an M2-stage lesion (a) and after complete clinical healing (M5-stage; b). (1) Clinical appearance of lesion site before (1a) and after (1b) treatment of 3 weeks duration. Circles highlight the biopsy sites. (2) Histological appearance of the biopsy of cow #13: (2a) Ulceration and extensive keratinolysis are evident in the stratum corneum. Rectangle shows selected area for in situ hybridization on a parallel section. H&E, bar 500 μm. (2b) The dermis is characterized by mild perivascular, chronic, lymphoplasmacytic dermatitis, without evidence of ulceration or signs of keratinolysis in the stratum corneum. H&E, bar 500 μm. (3) Double fluorescent in situ hybridization with probe for genus *Treponema* (Cy3 labelled) and for domain Bacterium (fluorescein labelled): (3a) Strong hybridization (score 3). *Treponema* organisms (arrows; orange) infiltrating deep into the epidermis, while other bacteria are visualized as green organisms (arrowheads); magnification (40x). (3b) No hybridization (score 0).

In total, 26 ulcerative M2-stage lesions diagnosed in 21 BDD-affected dairy cows were included in the study. The treatment duration ranged between two and four weeks (transition from M2-stage to M5-stage; **Table 1 and Fig 1**). None of the treated cows showed clinical signs of inflammation one week after B2 collection, and no BDD lesion reoccurred within one month after complete healing. Only two cows (#13 and #16) developed a proliferative and an ulcerative M2-stage lesion at 15 and 22 weeks after complete healing, respectively. Both cases were treated a second time as described above until M5-stage was reached. The duration of treatment of the recurrent lesions in these two cases was three weeks.

### Histopathological findings

**Table 1** provides a summary of observed histopathological changes in BDD lesions. All 12 M2-stage lesions showed evidence of ulceration and histopathological changes ranging from moderate to severe perivascular, chronic, lymphoplasmacytic dermatitis (**Fig 1**). In six M2-stage lesions, no spirochetes were detected by silver staining, while in the other six M2-stage cases, a low or moderate amount of spirochetes was found in the stratum corneum and deeper epidermal layers (**Table 1**).

In 6/26 M5-stage cases, no histopathological changes were seen, while 20 stage M5 lesions exhibited mild to moderate perivascular, chronic, lymphoplasmacytic dermatitis. All 26 M5-stage lesions were free from ulceration with mostly a keratinolysis score of 0 (**Table 1**).

In animals #13 and 16, lesions reoccurred and required a second round of treatment. The recurrent B1 lesion of animal #16 was ulcerated with a moderate perivascular, chronic, lymphoplasmacytic dermatitis and a moderate number of spirochetes with an extensive degree of keratinolysis. The following B2 biopsies of animals #13 and 16 were free from ulceration, no spirochetes were detected by silver staining and the keratinolysis score was 0 with a mild to moderate perivascular, chronic, lymphoplasmacytic dermatitis.

### TT PCR and sequencing results

Biopsies of the 12 randomly selected M2-stage and the 26 M5-stage lesions (one per animal) were screened for the presence of spirochetal DNA in a blinded manner. Following DNA extraction and successful β-actin PCR in all cases, M2- and M5-derived DNA isolates were subjected to consensus *Treponema* ("TT"-) PCR. From the 38 DNA extracts analyzed, 12/12 originating from M2-stage lesions, and 3/26 derived from M5-stage areas tested positive. TT PCR from positive, negative and no-template controls yielded anticipated results, thus confirming authenticity of obtained data. Four amplicons derived from M2-stage lesions of animals #2, 3, 5 (left hind leg), and 6 were 84–98% homologous to *T*. *phagedenis*. Another four amplicons originating from M2-stage lesions of cows #13, 14, 15, and 17 were 93–97% homologous to canine oral treponemes. The M2 affecting the hind left leg of cow #1 harbored a sequence sharing 85% identity with *T*. *medium* DNA. Two amplicons derived from M2-stage lesions of animals #4 and #5 were 94 and 91% homologous to *Treponema* clone PT4, and one amplicon originating from an M2 lesion affecting the right hind leg of animal #16 was 99% homologous to *Treponema* clone PT8.

The three amplicons of M5–derived DNA isolates of animals #11, 14, and 17 were 98, 98 and 87% identical to PT3, *T*. *medium* and *T*. *refringens*, respectively.

These data and further details including Genbank sequence accession numbers are provided in Table 1. M5-derived DNA isolates were different from the M2-derived DNA isolates of the cows #14 and #17 from which B1 biopsies were available. The M5–derived DNA isolates of re-treated animals #13 and 16 tested negative for *Treponema* spp.

### Results obtained by FISH

**Fig 2:**
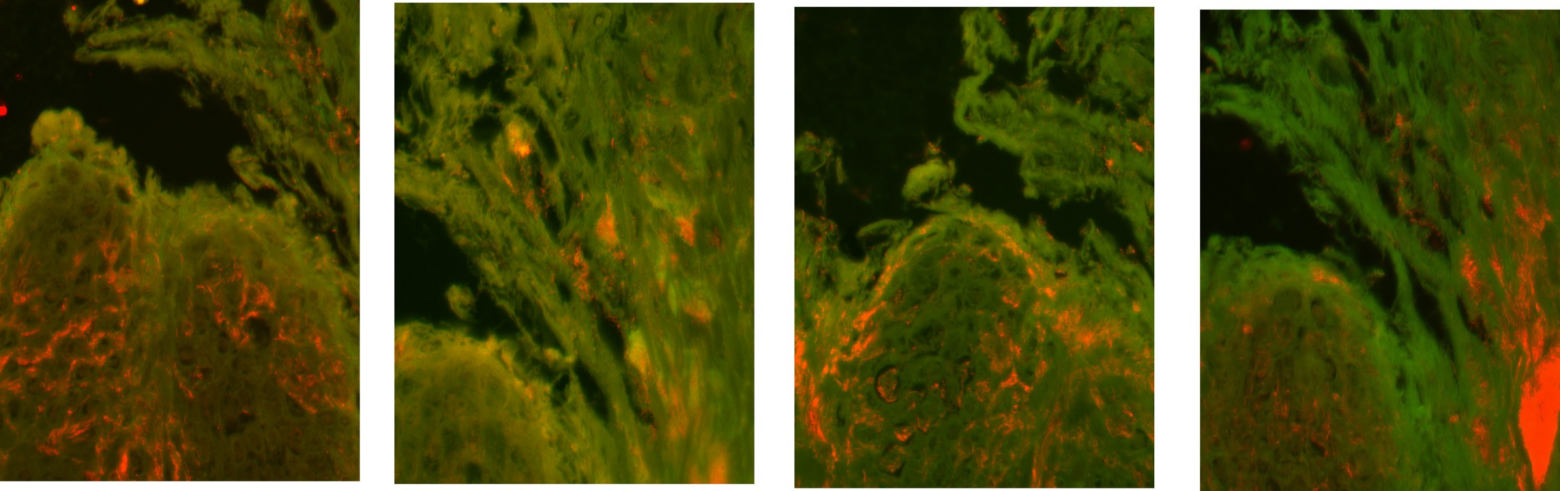
Identification of *Treponema* spp. infiltrating deep into the epidermis of a dairy cow (cow #13; age: 27 months) with ulcerative M2-stage lesions by fluorescent in situ hybridization. Four serial sections of the same biopsy hybridized with (Cy3 labelled) species specific oligonucleotide probes for A: *Treponema phagedenis*, B: *Treponema pedis*, C: *Treponema medium* and D: *Treponema refringens*. The *Treponema* organisms appear orange; magnification (40x).

Double in situ hybridization using the general oligonucleotide probe for the bacterial domain (eub-338) and the probe for *Treponema* spp. revealed treponemes infiltrating the epidermis in all 12 biopsies from M2-stage lesions (**Table 1 and Fig 1**). Severe, extensive treponemal epidermal infiltration (score 3) that constituted more than 90% of the total bacterial population was observed in all M2-stage biopsies. Infiltration of the dermis was rare and only observed superficially in ulcerations. Additional FISH analysis of *Treponema* spp.-positive M2-stage lesions with *T*. *phagedenis-*, *T*. *pedis*-, *T*. *medium*- and *T*. *refringens*-specific oligonucleotide probes revealed mixed, intermingled infections in all these lesions (**Fig 2**). *T*. *pedis* and *T*. *medium* phylotypes were found in all *Treponema* spp.-positive M2-stage lesions. Except for lesions of animal #6, *T*. *phagedenis* phylotypes were found in all *Treponema* spp.-positive M2-stage biopsies. Also *T*. *refringens* phylotypes were detected from *Treponema* spp.-positive M2-stage biopsies except for those of animals #15 and #16.

## Discussion

BDD is the most common infectious cause of severe, painful lameness in cattle, with anaerobic spirochetes of the genus *Treponema* having a major causative role in disease onset and progression. Topical application of antibiotics such as tetracycline in combination with footbaths are the current mainstay of BDD therapy [35]. Previous studies assessing the efficacy of topical tetracycline wrap and topical oxytetracycline application in BDD treatment resulted in complete clinical remission rates ranging from 9% [45] to 14–39% [46], respectively. In other studies, treatment success was defined as size reduction of the lesions or decreased pain reaction to palpation. Corresponding success rates ranged from 47–57% [30] to 68–73% [47] when using tetracycline hydrochloride bandages. Topical treatment with chlortetracycline resulted in a 79% reduction from M2-stage to non-M2 lesions per week [48]. To help reduce the use of antibiotics in veterinary medicine and by this, the risk of promoting resistances, recent studies have focused on evaluating the efficacy of alternative, non-antibiotic substances based on copper, zinc, iodine, or enzyme alginogel [16, 19, 29, 49–53]. However, none of these products was able to restore the M5-stage. In recent years, SA has emerged as promising nonantibiotic treatment option, and several studies have already shown that SA is effective in reducing or eradicating BDD lesions [18, 26, 28, 29]. Given that the therapeutic response to SA-based therapy was mainly determined by clinical examination, the impact of SA treatment on *Treponema* infection levels is still poorly understood. Histopathological evaluation of lesional biopsies in combination with clinical examination were only recently described, providing first insights into the performance of SA in relation to treponemal colonization [18].

Spontaneous BDD healing rarely occurs [54, 55]. M2-stage lesions usually persist for several months [56]. In the course of development of DD lesions, 94% of the transitions from healthy skin was observed as a transition finally ending up as hyperkeratotic lesions (M4-stage) and about 70% of the infected time was spent as M4-stage [57].

We opted for selective treatment of M2-stage ulcerative lesions (n = 26, >2 cm in cross section, affecting 21 dairy cows), because this stage is most painful, and hence, of utmost veterinary, economic and ethical relevance. In addition, M2-stage lesions represent a state of a finely tuned host-pathogen equilibrium that can persist for a long time and cannot be eliminated by application of local disinfectants [58]. Thus, adequate treatment of M2-stage lesions is essential to not only reduce pain and lameness, but also the pressure of infection, and the spread of *Treponema* spp. among healthy individuals [54].

Our optimized salicylic acid paste-based treatment concept consists of exposure of M2-stage lesions to SA paste under bandage for seven days per treatment round, repetition of treatment until complete disease resolution, and careful removal of the uppermost necrotic skin layer before every SA application proved to be essential for the therapeutic success. In general, the keratolytic effect of salicylic acid is superficial, affecting the stratum corneum and promoting epidermal repair, so that repeated treatment with SA under bandage is necessary to achieve clinical healing of M2-stage lesions. This particularly applies to lesions where deeper epidermal layers are invaded by *Treponema* spp. [11]. Moreover, *Treponema* spp. can persist in encysted forms that support chronicity, recurrence of infection and environmental persistence [6]. Using the herein described SA treatment protocol, repeated administration of SA under bandage at weekly intervals for two to four weeks resulted in complete clinical healing in 100% of cases independently of the severity and location of M2-stage lesions. Restored M5-stages exhibited no signs of ulceration or moderate to severe keratinolysis. In 2/21 animals, disease recurred after several months. Repeated treatment resulted in complete regression of the lesions and no further reoccurrence was observed during the study.

Repeated topical spray treatment of DD lesions with a 7.5% poly-vinyl-pyrrolidone-iodine complex or using PVP-iodine solution under bandage were both ineffective to treat DD lesions [59]. More recently, the topical administration of a combination of iodine solution with copper sulfate or honey showed only a short-term efficacy in terms of a transient reduction of lesion size [52]. Still, a positive effect of iodine preparations on the healing of DD lesions of our study cannot be excluded. For this reason, we abstained from pretreatment with povidone iodine ointment in 14/26 initial and one of two recurrent lesions. The results obtained in lesions with versus such without pretreatment with povidone iodine ointment were similar (median duration of treatment = 2 weeks; *P* = 0.8).

TT PCR proved to be the most sensitive method for detection of *Treponema* spp., followed by FISH and WS silver-staining. This finding was anticipated on the grounds that PCR constitutes a target DNA amplification method, which only requires the presence of a few target molecules to generate a positive result [60]. TT PCR from lesional DNA was carried out in a blinded manner, i.e. LM and SB processed enciphered samples. As anticipated, all M2-stage biopsies collected from 12/21 randomly selected BDD patients tested positive by TT PCR and FISH. Regarding *Treponema* phylotypes identified, TT PCR and FISH results diverged to some extent. This is due to the fact that PCR amplified the most abundant phylotype in the lesion, whilst FISH was designed for exclusive and simultaneous detection of *T*. *pedis*, *T*. *phagedenis*, *T*. *medium*, and *T*. *refringens*. However, where one of these four phylotypes was predominant in the lesion, TT PCR and FISH yielded concurrent results. Depending on the degree of infection, treponemes could also be visualized by silver-staining., i.e. in six of twelve M2-stage lesions harboring sufficient numbers of bacteria. These results are also in agreement with previous findings regarding the presence of different *Treponema* phylotypes in deeper epidermal layers flanking the dermal papillae [11, 44].

All M5-stage lesions tested consistently negative by WS silver-staining, and all except one also by FISH, indicating that treatment led to almost complete eradication of infection. The one FISH-positive M5-stage sample corresponded to one out of two biopsies taken from the same M5-stage, and harbored a small number of spirochetes located at the surface or outermost layers of the stratum corneum. The corresponding initial M2-stage lesion was ulcerative, affected the dorsal and plantar digital skin and also extended to the interdigital skin. Another aliquot of this FISH-positive M5-stage sample tested negative by TT PCR, suggesting that the low and superficial colonization detected by FISH constituted a local event within the stratum corneum.

Three of 21 M5-stage biopsy-derived DNA isolates scored positive by blinded TT PCR. Amplification products corresponded to PT3, *T*. *medium* and *T*. *refringens* DNA. This finding emphasizes the higher sensitivity of PCR in comparison to FISH, but is not necessarily indicative for persistence of infection in these three M5-stages, as the method cannot distinguish between viable and non-viable treponemes and DNA fragments thereof. Given that none of the three M5-stages progressed again to BDD within the observation time, it can be speculated that DNA from non-viable bacteria was amplified. As the most abundant *Treponema* spp. before and after treatment of these 2 cases were different, contamination in the course of biopsy sampling besides incomplete healing are further explanations.

Taken together, repeated application of SA paste under bandages at weekly intervals with careful removal of necrotic skin between treatment rounds allowed for complete clinical healing of M2-stage lesions in all 26 study cases. In combination with clinical and histopathological analyses, TT PCR and *Treponema* spp.-specific FISH provided consistent evidence that the presented SA treatment protocol is highly effective in eliminating *Treponema* infection not only from the stratum corneum, but also from deeper epidermal layers of initially ulcerative M2-stage lesions. Thus, complete clinical healing following the described treatment regimen is highly correlated with elimination of *Treponema* spp. To the authors’ knowledge, there is no DD treatment strategy of M2-stage lesions described in the literature with such a high (26/26) complete clinical healing rate.

## Conclusions

Repeated application of salicylic acid paste under bandages at weekly intervals allowed for complete clinical healing of M2-stage lesions in all 26 study cases. In combination with clinical and histopathological analyses, TT PCR and *Treponema* spp.-specific FISH provided sufficient evidence of elimination of *Treponema* spp. from epidermal layers underneath the stratum corneum of initially ulcerative M2-stage lesions. This outcome is highly promising and indicative of salicylic acid paste under bandage constituting an effective BDD treatment strategy.

## Supporting information

S1 Table Names and sequences of 16S rRNA-targeting oligonucleotide probes used in this study. (DOCX)

S1 Dataset Animals, cases used in this study and molecular biological analyses. (XLSX)
