# Supplementary material for: Proof of an optimized salicylic acid paste-based treatment concept of ulcerative M2-stage digital dermatitis lesions in 21 dairy cows
Source: PLoS One. 2022 Jun 9;17(6):e0269521. doi: 10.1371/journal.pone.0269521 (PMC9182225; doi:10.1371/journal.pone.0269521)
Supplement: S1 Table — (DOCX) [file pone.0269521.s001.docx]

**Table S1** Names and sequences of 16S rRNA-targeting oligonucleotide probes used in this study

| **Phylotype /**  **Species** | **Oligonucleotide probes** | **Sequence** | **Reference** |
| --- | --- | --- | --- |
| Bacteria | S-D-eub-338 | 5´-GTC ATT CCA TCG AAA CAT A-3´ | (1) |
| *Treponema* spp. | S-S-TrepGenus-725 | 5´-CAG AAA CYC GCC TTC GCC-3´ | (2) |
| *T. medium* | S-S-Trep-I:B:C7-432 | 5´-CAT CAG ATG AGC ATT CCC-3´ | (2) |
| *T. phagedenis* | PT6 | 5'-CA TCA AGG ACG CAT TCC CTC-3' | (3) |
| T. pedis | T. pedis | 5'-AG AGT CCT CAA CCT TTA CGT GTT-3' | (3) |
| *T. refringens* | Trep. refringens | 5'-GC TCC CTT TCC TTA CAT GAT-3' | (3) |

**Reference List**

1. **Amann RI, Ludwig W, Schleifer KH.** 1995. Phylogenetic identification and in situ detection of individual microbial cells without cultivation. Microbiol. Rev. **59**:143-169.
2. **Klitgaard K, Boye M, Capion N, Jensen TK.** 2008. Evidence of multiple *Treponema* phylotypes involved in bovine digital dermatitis as shown by 16S rDNA analysis and fluorescent *in situ* hybridisation. J. Clin. Microbiol. **46**:3012-3020.
3. **Rasmussen M, Capion N, Klitgaard K, Rogdo T, Fjeldaas T, Boye M, Jensen TK.** 2012. Bovine digital dermatitis: Possible pathogenic consortium consisting of *Dichelobacter nodosus* and multiple *Treponema* species. Vet. Microbiol. **160**:151-161.
